# Periapical and endodontic status among 65-year-old Oslo-citizens

**DOI:** 10.1186/s12903-022-02406-9

**Published:** 2022-09-01

**Authors:** My Tien Diep, Lene Hystad Hove, Dag Ørstavik, Rasa Skudutyte-Rysstad, Anne Thea Tveit Sødal, Pia Titterud Sunde

**Affiliations:** 1 Department of Cariology and Gerodontology, Faculty of Dentistry, University of Oslo, Blindern, P.O. Box 1109, N-0317 Oslo, Norway; 2 Department of Endodontics, Faculty of Dentistry, University of Oslo, Oslo, Norway

**Keywords:** Aged, Epidemiology, Apical periodontitis, Endodontics

## Abstract

**Aim:**

This cross-sectional study aimed to investigate the prevalence of apical periodontitis (AP) and root-filled teeth in a 65-year-old population in Oslo, Norway, and to investigate associations of pathosis and endodontic treatment with selected individual risk indicators and technical quality of root fillings.

**Material and methods:**

A random sample of 450 65-year-olds in Oslo answered a questionnaire and underwent a clinical and radiological examination (52% men and 48% women). Periapical radiographs were taken of all root-filled teeth and of teeth with apical radiolucency, and periapical status was evaluated using the Periapical Index. Apex-to-filling distance and homogeneity were assessed for all root fillings. Analyses on individual level and tooth level were performed. The outcome variables were ‘non-root-filled tooth with AP’ (‘untreated AP’), ‘root-filled tooth’, and 'root-filled tooth with AP’. The explanatory variables were gender, education, dental attendance pattern, smoking, remaining teeth (n), tooth group, and root filling quality. Chi-square test and logistic regression analyses were used to assess the associations between outcome variables and explanatory variables. The level of significance was set to *p* < 0.05.

**Results:**

The mean number of remaining teeth was 26 (SD: 4). AP was present in 45% of the individuals. Sixteen percent of the individuals had untreated AP and 38% had at least one root-filled tooth with AP. Sixty-six percent of the individuals had one or more root-filled teeth. Untreated AP was significantly associated with a decreasing number of remaining teeth and smoking. All the outcome variables were significantly more prevalent in molars compared with premolars and anterior teeth. Thirty-five percent of the root-filled teeth had AP, and AP was more prevalent in teeth with too short apex-to-filling distance (53%) or unsatisfactory homogeneity (46%).

**Conclusions:**

The remaining number of teeth was high, and AP and root-filled teeth were prevalent in the present young-elderly population. A notable amount of untreated AP was observed, especially in smokers. The findings in the present study indicate a substantial need for dental care associated with endodontic conditions in the future elderly.

**Supplementary Information:**

The online version contains supplementary material available at 10.1186/s12903-022-02406-9.

## Introduction

Periapical and endodontic conditions influence tooth survival [1]. From a public health perspective, it is therefore important to map these conditions in the young-elderly in order to plan for future dental health needs and services in an aging population. The beginning of old age is often defined as 65 years of age, although there is no general agreement [2, 3].

Apical periodontitis (AP) is inflammation and destruction of periapical tissues caused by endodontic infection [4, 5]. The infection is treated by root canal disinfection and subsequent root-filling, endodontic microsurgery or tooth extraction [6]. Researchers have pointed out that in the elderly, endodontic treatment may become more difficult to perform due to potentially previous treatment traumas and age changes to the pulp [7]. The fact that aging is correlated with reduced general health can also impose challenges for endodontic treatment, as elderly, fragile patients may find prolonged dental visits exhausting.

During the last two decades, several studies on the prevalence of AP and root-filled teeth have been performed [8–22], but few have studied random samples recruited from the general population [9, 11, 14, 18, 19, 22], and even fewer have focused on the young-elderly. A recent meta-analysis showed that the frequency of AP was higher in individuals recruited from dental care services and hospitals than from the general population [23]. Moreover, several studies on random samples from the general population [24–28] were carried out more than 20 years ago, which may limit their representativeness of the present young-elderly population.

Results from a systematic review [29] found a higher prevalence of AP and endodontically treated teeth among subjects older than 50 years. Previous studies investigating the relationship between periapical pathosis and socioeconomic and behavioural variables found that AP was statistically associated with smoking status [30], level of education [31] and regularity of dental attendance [30, 31]. In addition, the association between the number of remaining teeth and AP has previously both been confirmed [30] and disconfirmed [32]. However, these studies did not differentiate between AP in endodontically treated and untreated teeth, which may not be associated with the same determinants, as demonstrated in the longitudinal study by Kirkevang & co-workers [1]. For instance, tooth-specific factors, such as poor technical quality of the root filling, are strong risk indicators for AP in root-filled teeth [17, 21, 33].

The aims of this study were therefore to investigate the prevalence of AP and root-filled teeth among 65-year-olds in Oslo, Norway, and to investigate associations of pathosis and endodontic treatment with selected individual risk indicators and technical quality of root fillings.

## Material and methods

### Study design and setting

The present cross-sectional study was part of a larger study investigating oral health in 65-year-olds in Oslo, Norway (the OM65 study). As previously described [34], the participants were examined by two dentists (MTD and ATTS) at the Research Clinic, Institute of Clinical Dentistry, University of Oslo, between February and December 2019. The study protocol was approved by the Norwegian Regional Committee for Research Ethics (REK 2018/1383) and the study was performed in compliance with the tenets of the Declaration of Helsinki. All participants signed a written informed consent form. The present paper was written using the ‘strengthening the reporting of observational studies in epidemiology’ (STROBE) guidelines.

### Participants

The study population was 65-year-old (born in 1954) residents of Oslo, Norway. Accounting for the possibility of a longitudinal follow-up study after 5 years, the calculated sample size was 450 participants. Eligible individuals were randomly selected from the National Population Register, which is administered by the Norwegian Tax Administration authorities, and invitation letters were sent to 1230 individuals. Within 2 weeks, the individuals were called and asked if they were interested in participating in the study.

### Questionnaire

A self-administered questionnaire was sent to the participants via an electronic link to an online questionnaire program (Nettskjema, University of Oslo). Participants answered the questionnaire prior to attending the clinical examination. The participants were asked about smoking status (never, former or current), highest level of completed education, and dental attendance pattern. The participants’ level of education was dichotomised into ‘higher education’ (university/college) and ‘basic education’ (high school, elementary school, or lower). Dental attendance pattern was dichotomised into ‘regular’ and ‘irregular’ (occasionally, only emergency visits, or never).

### Radiographic examination

Panoramic radiographs (orthopantomogram, OPG) were taken of all participants (ProMax X-ray Dimax 3 and Planmeca ProOne, Planmeca Oy, Helsinki). Dentists, working at the Department of Maxillofacial Radiology at the Faculty of Dentistry, University of Oslo, screened the OPGs, and ordered additional periapical radiographs of all the root-filled teeth and of the teeth with apical radiolucency. These radiographs were taken with an intraoral imaging unit (Focus, KAVO, Instrumentarium Dental, PaloDEx Group Oy, Tuusula, Finland) with a rectangular collimator (length 30.5 cm, radiation area 35 × 45 mm), exposure 70 kV, 7 mA, 0.10–0.16 s, using the paralleling technique and intraoral phosphor plates (DIGORA Accessory intraoral imaging plates, Soredex Tuusula, Finland).

### Radiographic registration methods

Table 1 describes the parameters assessed from the periapical radiographs. Periapical status was evaluated for root-filled teeth and teeth with AP using the periapical index (PAI) [35]. In cases of multi-rooted teeth, the score of the root with the highest PAI score was used. AP was defined as PAI score > 2. The distance from the radiographic apex to the root filling was also measured. For multi-rooted teeth, the largest apex-to-filling distance among the roots (over- and under-filling treated equally) was used, independently of the PAI score and homogeneity. The registration categories were as defined by Kirkevang and co-workers [7]. Homogeneity of the root filling was evaluated using the visual scoring system developed by Jordal and co-workers [36]. For multi-rooted teeth, the highest homogeneity score among the roots was used, independently of the PAI score and apex-to-filling distance. Table 1 also describes the categorisations used for statistical analyses. The radiographs were assessed using the ImageJ software [37].

**Table 1:** Radiographic parameters for periapical status and root-filling quality

Parameters	Registration category	Analysis category
Periapical index (PAI)^a^	1 = Normal periapical structures	Not pathological = 1 + 2 Pathological = 3 + 4 + 5
	2 = Small changes in bone structure	
	3 = Changes in bone structure with some mineral loss	
	4 = Periodontitis with well-defined radiolucent area	
	5 = Severe periodontitis with exacerbating features	
Apex-to-filling distance^b^	1 = Root filling ending ≤ 3 mm from radiographic apex	Satisfactory = 1 + 4 Short = 2 + 3 Long = 5
	2 = Root filling ending > 3 mm from radiographic apex	
	3 = Pulpotomy, material seen only in the pulp chamber	
	4 = Flush, root filling ending at the radiographic apex	
	5 = Over-filling, root filling material seen in the periapical area	
Homogeneity of root filling^c^	1 = Homogeneous root filling	Satisfactory = 1 Unsatisfactory = 2 + 3 + 4
	2 = Minor irregularities in the root filling	
	3 = Voids in the root filling	
	4 = Larger voids in the root filling	

^a^Ørstavik et al*.* 1996

^b^Kirkevang et al*.* 2000

^c^The description of the registration categories is based on the visual scale for evaluation of root filling quality published by Jordal et al*.* 2014

### Observer

One observer evaluated all periapical radiographs (MTD). The observer was prepared for using the PAI system by calibration against a reference set of 100 radiographic images of teeth. The weighted kappa value was 0.73 (95% CI 0.71–0.77) for reproducibility compared to the reference set and 0.72 (95% CI 0.69–0.75) for intra-observer reproducibility. The same calibration procedure has been described in more detail by Kirkevang and co-workers [38]. In cases of doubt, the radiographs were discussed with an endodontist (PTS), in order to achieve a consensus.

### Statistical analyses

Registrations were entered in the Oral Data Collector (ODC) sheet specifically designed for data entry in this study, developed in Microsoft Excel 2016 (Microsoft Corporation, Redmond, Washington, USA), processed using openpyxl 3.0.4 and pandas 1.1.0 in Python 3.8 (Python Software Foundation, https://www.python.org/) and imported into STATA (Stata version 16.1; College Station, TX, USA) for statistical analyses. Descriptive statistical analyses were performed and the results are presented in the form of numbers (n) with percentages (%) and mean with standard deviation (SD). The outcome variables were ‘untreated AP’ (non-root-filled teeth with AP), ‘root-filled’, and ‘root-filled with AP’ on both individual level and tooth level, and the prevalence on individual level was defined as having at least one tooth with the respective condition. The chi-square test was used to determine significant differences in the prevalence of periapical and endodontic conditions related to gender, education level, dental attendance pattern, and smoking status (categorical variables). For continuous explanatory variables (number of remaining teeth), univariate logistic regression was used to assess the associations with the outcome variables. Since untreated AP showed a significant association with the highest number of socioeconomic and behavioural variables, this relationship was further explored using univariate and multivariate logistic regression. In all cases of multivariate logistic regression, the analyses were only adjusted for the variables shown in the respective table. For the analyses on tooth level, generalised estimating equation for logistic regression was used, accounting for possible correlations between the teeth belonging to the same individual (dependence within clusters). The results from the regression analyses are presented as unadjusted and adjusted odds ratios with a 95% confidence interval. The level of significance was set to *p* < 0.05. The data were securely stored and the analyses were performed in Service for Sensitive Data (TSD), Centre for Information Technology Services, University of Oslo.

## Results

Of the 797 eligible participants who were reached by telephone after having received the invitation letter, 460 attended the clinical and radiographic examination (response rate of 58%). Ten individuals were excluded from the analyses due to missing periapical radiographs or low quality of the radiographs. Thus, the final sample comprised 450 individuals. However, three individuals did not answer the questionnaire, resulting in self-reported data from 447 individuals. The sample population was characterised by an almost even gender distribution (52% men, 48% women), and a predominance of those with higher education (66%), regular dental attenders (89%), and non-smokers (89%) (Table 2). The mean number of teeth per individual was 26 (SD: 4, range: 0–28) and 94% of the individuals had more than 20 remaining teeth. Two individuals were edentulous.

**Table 2:** Untreated AP, root-filled teeth and root-filled teeth with AP according to gender, level of education, smoking and dental visiting habits

		No. of individuals (%)	At least one tooth with untreated AP n (%)	At least one root-filled tooth n (%)	At least one root-filled tooth with AP n (%)
All*		447 (100)	72 (16)	296 (66)	170 (38)
*Gender*					
	Male	232 (52)	44 (19)	162 (70)	96 (41)
	Female	215 (48)	28 (13)	134 (62)	74 (34)
*Education*					
	Higher education	297 (66)	39 (13)^a^	198 (67)	113 (38)
	Basic education	150 (34)	33 (22)^a^	98 (65)	57 (38)
*Dental attendance*					
	Regular	398 (89)	59 (15)^a^	272 (68)^a^	149 (37)
	Irregular	49 (11)	13 (27)^a^	24 (49)^a^	21 (43)
*Smoking*					
	Never	193 (43)	25 (13)^a^	113 (59)^a^	65 (34)
	Former	205 (46)	30 (15)^b^	151 (74)^a^	82 (40)
	Current	49 (11)	17 (35)^ab^	32 (65)	23 (47)

AP Apical periodontitis

*Missing data because three individuals did not answer the questionnaire

Letters indicate a statistically significant difference between groups of the same letter within the same variable (*p* < 0.05: Chi-square)

### Individual level

AP was present in 45% of the individuals. Sixteen percent of the individuals had at least one tooth with untreated AP and 38% had at least one root-filled tooth with AP. Sixty-six percent of the individuals had one or more root-filled teeth. Ten percent of the individuals had three or more teeth with AP, and 25% of the individuals had three or more root-filled teeth. The distribution of individuals according to the total number of teeth with AP, untreated AP, root-filled teeth, and root-filled teeth with AP may be seen in Additional file 1: Table S1.

#### Number of remaining teeth

Table 3 shows the distribution of untreated AP, root-filled teeth, and root-filled teeth with AP by number of remaining teeth. Untreated AP was significantly associated with decreasing number of remaining teeth (logistic regression: *p* < 0.05) [Additional file 1: Table S2].

**Table 3:** Untreated AP, root-filled teeth and root-filled teeth with AP according to number of remaining teeth

	No. of individuals (%)	At least one tooth with untreated AP n (%)	At least one root-filled tooth n (%)	At least one root-filled tooth with AP n (%)
*All No. of remaining teeth*	450 (100)	72 (16)	296 (66)	169 (38)
0	2 (0.4)	–	–	–
1–5	4 (0.9)	2 (50)	1 (25)	1 (25)
6–10	0 (0)	–	–	–
11–15	7 (2)	1 (14)	6 (86)	4 (57)
16–20	14 (3)	5 (36)	8 (57)	5 (36)
21–25	126 (28)	25 (20)	87 (69)	53 (42)
26–28	297 (66)	39 (13)	195 (66)	107 (36)

*AP* Apical periodontitis

#### Socioeconomic and behavioural variables: bivariate analyses

Table 2 sums up the results from the bivariate analyses on the prevalence of untreated AP, root-filled teeth, and root-filled teeth with AP on individual level according to gender, level of education, smoking status, and dental attendance pattern. Untreated AP was significantly associated with the level of education, dental attendance pattern, and smoking status; it was more prevalent in those with basic education compared to those with higher education (22 vs. 13%), in irregular dental attenders compared to regular dental attenders (27 vs. 15%), and in current smokers (35%) compared to former smokers (15%) and never-smokers (13%). Furthermore, root-filled teeth were significantly more prevalent in former smokers compared to never-smokers (74 vs. 59%) and in regular dental attenders compared to irregular dental attenders (68 vs. 49%). No statistically significant associations were found between the socioeconomic and behavioural variables and the prevalence of root-filled teeth with AP.

#### Socioeconomic and behavioural variables: logistic regression

Basic educational level, irregular dental attendance, and current smoking were significantly associated with the presence of at least one tooth with untreated AP in the unadjusted analyses (Table 4). However, current smoking was the only significant risk indicator in the adjusted analyses and was associated with a threefold increase in odds ratio of having untreated AP.

**Table 4:** Exploratory logistic regression model: untreated AP and socioeconomic and behavioural factors

N = 447 Explanatory variables	Unadjusted odds ratio (95% CI)	Adjusted odds ratio (95% CI)
Gender		
Female	1	1
Male	1.6 (0.9–2.6)	1.4 (0.8–2.5)
Education level		
Higher education	1	1
Basic education	**1.7 (1.1–3.1)**	1.6 (0.9–2.7)
Dental visits		
Regular	1	1
Irregular	**2.1 (1.0–4.1)**	1.5 (0.7–3.2)
Smoking		
Never	1	1
Former	1.2 (0.7–2.0)	1.1 (0.6–2.0)
Current	**3.6 (1.7–7.4)**	**3.0 (1.4–6.4)**

*CI* Confidence interval

Outcome variable: at least one tooth with untreated AP

Values shown in bold text differ significantly (*p* < 0.05: Logistic regression) from the reference category

### Tooth level

**Fig. 1:**
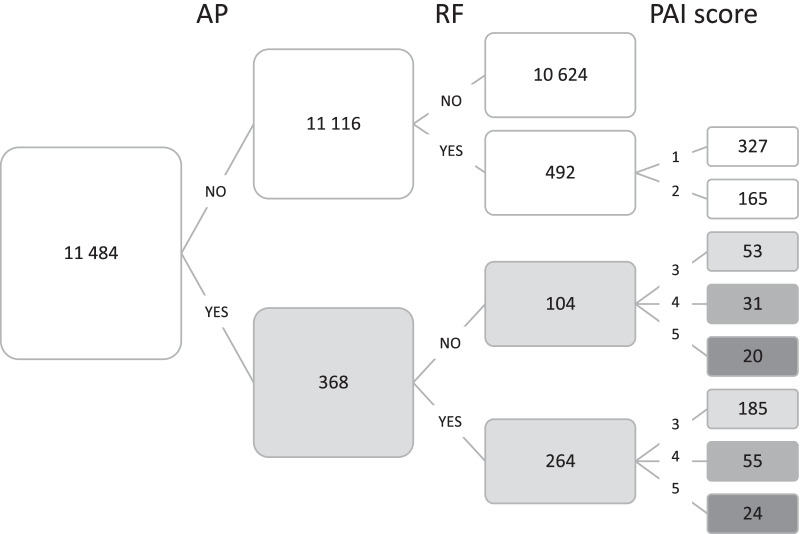
Distribution of number of teeth (N = 11 484) according to AP, RF and PAI. AP = apical periodontitis; RF = root filled; PAI = Periapical Index

Figure 1 shows the distribution of the total number of teeth examined (N = 11,484) according to periapical and endodontic status. In total, 368 teeth (3%) had AP, and 28% of these were not root-filled. The total number of root-filled teeth was 756 (7%), of which 35% had AP.

#### Tooth group and jaw

The distribution of untreated AP, root-filled teeth, and root-filled teeth with AP in tooth groups divided by jaw is shown in Table 5. The outcome variables were more strongly associated with molars compared with premolars and anterior teeth (logistic regression: *p* < 0.05). Maxillary premolars and molars had higher odds of having untreated AP compared to the same tooth groups in the mandible (logistic regression: *p* < 0.05). This difference was not found for anterior teeth. Maxillary anterior teeth and premolars had higher odds of being root-filled or having AP related to a root filling compared to the same tooth groups in the mandible (logistic regression: *p* < 0.05). This difference was not found for molars.

**Table 5:** Distribution of untreated AP, root-filled teeth and root-filled teeth with AP in tooth groups stratified by jaw

	No. of teeth (%)	Untreated AP n (%)	RF n (%)	RF with AP n (%)
Total	11,484 (100)	104 (0.9)	756 (7)	263 (2)
Maxillary				
Anterior teeth	2601 (23)	10 (0.4)	110 (4)	33 (1)
Premolars	1542 (13)	21 (1.4)	154 (10)	49 (3)
Molars	1549 (13)	33 (2.1)	176 (11)	85 (5)
Mandibular				
Anterior teeth	2623 (23)	12 (0.5)	30 (1)	7 (< 1)
Premolars	1608 (14)	9 (0.6)	101 (6)	18 (1)
Molars	1561 (14)	19 (1.2)	185 (12)	72 (5)

*AP* Apical periodontitis, *RF* root-filled

#### Quality of root filling

In total, 76% of the root fillings were satisfactory in the apex-to-filling distance, 57% were satisfactory in homogeneity and 48% were satisfactory in both apex-to-filling distance and homogeneity (Table 6). AP was significantly associated with teeth with too short root fillings or unsatisfactory homogeneity (logistic regression: *p* < 0.05) [Additional file 1: Table S3].

**Table 6:** Periapical status according to root filling quality

N = 756	No. of root filled teeth (%)	No AP n (%)	AP n (%)
*Apex-to-filling distance (ATF)*			
Satisfactory	575 (76)	401 (70)	174 (30)
Short	142 (19)	67 (47)	75 (53)
Long	39 (5)	24 (62)	15 (38)
*Homogeneity*			
Satisfactory	434 (57)	319 (74)	115 (27)
Unsatisfactory	322 (43)	173 (54)	149 (46)
*ATF & homogeneity*			
Satisfactory	361 (48)	269 (75)	92 (25)
Unsatisfactory*	395 (52)	223 (56)	172 (44)

*AP* Apical periodontitis

*Unsatisfactory apex-to-filling distance, homogeneity or both

## Discussion

This paper describes the prevalence of apical pathology and root-filled teeth in a random sample of 65-year-old Oslo-citizens, and explores the associations of pathosis and treatment with selected individual risk indicators and the technical quality of endodontic treatment. To our knowledge, the present study is one of few studies that focus on these conditions in a young-elderly population.

The results demonstrate that AP and root-filled teeth were prevalent in the present sample population. This and the fact that the present young-elderly population has retained most of their own teeth indicates that many of these individuals may be at risk of dental problems when facing old age.

In Norway, dental services are divided into a public and private sector [39, 40]. Dental treatment provided by the public dental service is free for patients 0–18 years of age, mentally handicapped adults and elderly living in an institution or receiving home nursing care. The majority of adults receive dental care from private general dental practitioners, mainly financed by patient charges. Thus, the dental treatment experience of 65-year-olds in Norway will mainly be based on self-financed treatments in the private sector. Certified endodontists also mainly work in the private sector, but the proportion of patients or teeth receiving specialist treatment is unknown.

### Comparison of prevalence estimates

AP and root-filled teeth are more prevalent in older compared to younger age groups [38]. This is confirmed by comparing the findings from the present study with previous findings in 35-year-old Oslo-citizens [9]. Therefore, the present study sought to compare the present prevalence estimates with findings in similar age groups if such data were available. Table 7 lists a selection of studies on periapical and endodontic conditions, which are considered comparable to the present study due to similar recruitment procedures and the fact that they were performed during the last two decades. All the listed studies evaluated the periapical status on periapical radiographs and defined AP as a PAI score > 2, except for one study [19], which registered AP in panoramic radiographs and defined AP as the periodontal ligament being two times the normal width.

**Table 7:** Previous studies on periapical and endodontic conditions in the general population conducted after year 2000

Country	Author	Exam year	No. of individuals	No. of teeth	Age range	Comparable age group (n)	Individual level		Tooth level			
							AP/total (%)	RF/total (%)	AP/total (%)	Untreated AP/total (%)	RF/total (%)	RF with AP/RF (%)
Norway	Diep et al. (present study)	2019	450	11 484	65	–	45	66	3,2	0,9	6,6	35
Denmark	Razdan et al. (2022)	2009	398	10 668	20–64	60–64 (109)	68,8*	67,9*	5,5*	2,4*	5,6*	57,0*
Denmark	Kirkevang et al. (2012)	2008	360	9 350	30–72	–	53	59	4,2	–	5,8	42,9
Sweden	Virtanen et al. (2017)	2003	120	–	49–58	49–58	40,8*	60,8	–	–	–	–
Sweden	Frisk et al. (2008)	2003	491	12 433	20–70	–	–	–	–	–	–	24,8
Norway	Skudutyte et al. (2006)	2003	146	3 971	35	–	16	23	1,1	0,4	1,5	43
Finland	Huumonen et al. (2017)**	2001	5335	120 635	30–95	55–64 (929)	34*	67*	–	–	6,6	15,3

*AP* apical periodontitis, *RF* root-filled

*Results in comparable age group

**Registered AP in panoramic radiographs and defined AP as the periodontal ligament being two times the normal width

The prevalence of AP in the present population sample (45%) was somewhat higher compared to findings in a similar age group in Finland (34%) [19] and Sweden (41%) [18], but lower compared to results from Denmark (69%) [22]. Unlike the present study, separate prevalence estimates for untreated AP and root-filled teeth with AP on individual level were not reported in any of the papers listed in Table 7. However, on tooth level, Razdan and co-workers found a 2,sixfold higher prevalence of untreated AP compared to the present study [22]. Furthermore, the prevalence of AP in root-filled teeth in the present study (35%) was within the range of the results from the comparable studies (15–57%) [9, 11, 14, 19, 22].

Sixty-six percent of the individuals in the present study had one or more root-filled teeth, which is in line with previous reports (61–68%) [18, 19, 22].

The variation among the populations examined, for example in age distribution and socioeconomic status, can probably explain some of the discrepancies between the different prevalence estimates. In addition, differences in study designs regarding inclusion criteria, types of radiographs, and definition of AP may have influenced the results, as well as possibly different treatment routines between the countries. In the present study, the intention of not excluding edentulous individuals was that the prevalence estimates were meant to represent the whole target population, not only the dentate population. However, it did not notably affect the results, since only 0.4% of the present sample were edentulous.

### Number of remaining teeth

The presence of untreated AP was associated with a decreasing number of remaining teeth. This may indicate that the missing teeth were mainly lost or extracted for other reasons than AP, such as periodontitis or dental trauma. It can also be speculated that the incidence of caries and subsequent AP is higher in individuals with fewer remaining teeth, without a matching treatment rate, and therefore the undergone extractions have not resulted in a lower prevalence of AP compared to individuals with more remaining teeth. However, longitudinal studies are needed to provide further insight related to this hypothesis. Nevertheless, it is important to underline that, in the present study, only 11 individuals had 1–15 teeth, hence the results should be interpreted with caution.

### Gender

In accordance with previous studies [38, 41], there was not a significant difference in the presence of AP between the genders. Other studies have reported both that the presence of AP was more prevalent in men [11, 19], and in women [18]. In addition, the present study did not find a significant gender difference in the prevalence of root-filled teeth, unlike other studies that found that it was more prevalent in women [42].

### Dental attendance pattern

In the present study, having at least one root-filled tooth was more frequent among regular dental attenders compared with irregular dental attenders. This indicates that regular dental attenders are more subjected to conservative treatment (e.g. endodontic treatment) compared with irregular dental attenders who may be more subjected to emergency treatment (e.g. tooth extractions) or no treatment.

### Smoking

The presence of untreated AP was associated with basic educational level, irregular dental attendance, and current smoking in the bivariate analyses. However, only current smoking was significant in the multivariate regression analysis. This indicates that among individuals with the same smoking status, education and dental attendance pattern did not significantly affect the outcome. Smoking may increase the risk of developing caries [43], followed by a possible increased risk of AP and endodontic treatment. In the present study, the presence of root-filled teeth was more frequent in former smokers than never-smokers.

Previous reports have shown an association between AP and smoking [18, 30]. The authors explained this observation with potentially delayed bone healing in smokers compared to non-smokers. However, since untreated AP cannot be in a healing process, this can only be a reasonable explanation for AP related to root-filled teeth. Nonetheless, in accordance with previous findings [32], no significant association was found between root-filled teeth with AP and smoking status in the present study. This indicates that although delayed bone healing may be present in smokers, once the AP has been treated, other determinants, such as the treatment quality, may be more relevant. A previous study showed that after accounting for the quality of root filling, smoking was no longer associated with AP [44].

### Tooth group and jaw

Overall, in the present study, the prevalence of endodontically treated and untreated AP was higher in molars compared with the other tooth groups. Achieving satisfactory root filling quality in molars compared to premolars and anterior teeth can be more challenging due to multiple roots and more complicated root canal anatomy, especially in maxillary first molars. Huumonen and co-workers found that only 26% of root fillings in maxillary molars and 34% of root fillings in mandibular molars had satisfactory apex-to-filling distance [42].

In the present study, mandibular molars were the tooth group most frequently root-filled (12%), in line with a study from Finland (12%) [42]. However, in the same study, twice as many maxillary anterior teeth were root-filled compared to the present study (8% vs. 4%).

Maxillary anterior teeth and premolars had a higher risk of being root-filled and having a root filling with AP compared with mandibular anterior teeth and premolars. This pattern may be due to more dental caries and trauma in the maxillary compared to the mandibular anterior teeth. Moreover, it has been shown in children 5–16 years old that maxillary premolars are more susceptible to caries than mandibular premolars, which may also apply for adults [45].

### Quality of root fillings

Previous studies have found that the periapical outcome is strongly influenced by the quality of the endodontic treatment [11, 19]. In the present study, both too short apex-to-filling distance and voids in the root filling were associated with the presence of AP. These findings confirm previous reports, in which some have only looked at the apex-to-filling distance [19, 42], whereas others have reported both apex-to-filling distance and homogeneity separately [7, 11] or combined [24, 31, 46]. In the present study, 76% of the root fillings had satisfactory apex-to-filling distance, compared to 47% in a Finish study [42]. Despite this, the occurrence of AP in root-filled teeth in the present study was higher than in the Finish study (35% vs. 15%). The fact that there was a higher root-filled molars/root-filled anterior teeth ratio in the Finish study (33%/32%) compared to the present study (48%/19%) may partly explain this paradoxical observation, given that endodontic treatment has a better success rate in anterior teeth compared to molars, as previously discussed. In the present study, having root-filled teeth with AP was not associated with any of the socioeconomic or behavioural variables.

### Study limitations

The present study has potential limitations. The attendance rate in this study was 58%, which is comparable to previous studies with similar recruitment procedures (51–64%) [9, 38]. Due to restrictions from the Ethics Committee, we were not permitted to ask non-attenders why they declined to participate. Therefore, in order to explore potential selection bias, the gender distribution and education level of the sample population were compared with the corresponding proportions of the study population (based on register data on 65-year-olds living in Oslo, from Statistics Norway). The gender distribution was similar, but the proportion with higher education in the current sample population was higher than the average in the study population. This may have affected the prevalence estimates; untreated AP was more prevalent in those with only basic education, which means that the prevalence of untreated AP in the present study may have been underestimated.

A self-administered questionnaire was used to assess smoking status and dental attendance patterns. Therefore, these data are dependent on the responder's interpretation of the questions and their ability to recall or identify the requested information, potentially resulting in recall bias.

Cross-sectional studies cannot distinguish between healing and progressing cases of AP in root-filled teeth. Some studies have demonstrated that elapsed time plays an important role in the healing of AP [14], while other results suggest that this is not a major issue in large epidemiological studies, since the proportion of developing lesions (not detectable in radiographs) and healing lesions are similar [26].

The success rate of endodontic treatment is influenced by the periapical status at the start of treatment; root-filling of a non-infected tooth (vital pulp extirpation) has a better prognosis than root-filling of an infected tooth (treatment of AP) [47]. Another potential distorting factor may therefore be the missing information about the pre-treatment diagnosis, e.g. optimal apex-to-filling distance of the root filling is probably less determining in teeth without preoperative AP [48].

Periapical radiographs are more sensitive to the detection of periapical lesions than panoramic radiographs [49]. In the present study, only non-root-filled teeth suspected of having AP in panoramic radiographs were selected for further evaluation using periapical radiography. This may have led to an underestimation of untreated AP. Furthermore, 3D radiographic examinations, such as cone beam computed tomography (CBCT), may be more sensitive in detecting periapical pathology compared to 2D radiographic examinations. However, the latter retains an effective validity [50, 51], inflicts less radiation to the study participants and are less time consuming, all beneficial factors in larger observational studies.

## Conclusions

The remaining number of teeth was high, and AP and root-filled teeth were prevalent among 65-year-olds in Oslo. A notable amount of untreated AP was observed, especially in current smokers. Approximately one-third of the root-filled teeth had AP; this was significantly more common if the apex-to-filling distance was too short or if the root filling was not homogeneous. The findings in this young-elderly population indicate a substantial need for dental care associated with endodontic conditions in the future elderly.

## Supplementary Information

**Additional file 1: Supplementary Table 1:** Distribution of individuals (N = 450) by frequency of AP, untreated AP, RF teeth and RF teeth with AP. **Supplementary Table 2:** Associations of untreated AP, root-filled teeth and root-filled teeth with AP with number of remaining teeth (n). **Supplementary Table 3:** The association between apical periodontitis and quality of root filling.

## Data Availability

The data that support the findings of this study are available on request from the corresponding author. The data are not publicly available due to privacy or ethical restrictions.
