# Supplementary material for: Periapical and endodontic status among 65-year-old Oslo-citizens
Source: BMC Oral Health. 2022 Sep 1;22:371. doi: 10.1186/s12903-022-02406-9 (PMC9438292; doi:10.1186/s12903-022-02406-9)
Supplement: Supplementary file 1 — Additional file 1: Supplementary Table 1: Distribution of individuals (N = 450) by frequency of AP, untreated AP, RF teeth and RF teeth with AP. Supplementary Table 2: Associations of untreated AP, root-filled teeth and root-filled teeth with AP with number of remaining teeth (n). Supplementary Table 3: The association between apical periodontitis and quality of root filling. [file 12903_2022_2406_MOESM1_ESM.docx]

| **Supplementary Table 1.** Distribution of individuals (N = 450) by frequency of AP, untreated AP, RF teeth and RF teeth with AP | | | | |
| --- | --- | --- | --- | --- |
| Frequency | AP | Untreated AP | RF | RF with AP |
|  | n (%) | n (%) | n (%) | n (%) |
|  |  |  |  |  |
| 0 | 249 (55) | 378 (84) | 153 (34) | 280 (62) |
| 1 | 115 (26) | 52 (12) | 101 (22) | 109 (24) |
| 2 | 42 (9) | 11 (2) | 82 (18) | 42 (9) |
| 3 | 23 (5) | 8 (2) | 47 (10) | 12 (3) |
| 4 | 14 (3) | 0 (0) | 26 (6) | 4 (<1) |
| 5 | 4 (1) | 0 (0) | 14 (3) | 2 (<1) |
| 6 | 1 (<1) | 1 (<1) | 18 (4) | 0 (0) |
| 7 | 0 (0) | 0 (0) | 5 (<1) | 0 (0) |
| 8 | 1 (<1) | 0 (0) | 3 (<1) | 0 (0) |
| 9 | 1 (<1) | 0 (0) | 1 (<1) | 1 (<1) |
|  |  |  |  |  |
| AP = apical periodontitis; RF = root-filled | | |  |  |

| **Supplementary Table 2.** Associations of untreated AP, root-filled teeth and root-filled teeth with AP with number of remaining teeth (n) | | | | | |
| --- | --- | --- | --- | --- | --- |
| N = 11 484 | | Untreated AP | RF | | RF with AP |
|  |  | Odds ratio (95% CI) | Odds ratio (95% CI) | | Odds ratio (95% CI) |
|  |  |  |  | |  |
| Number of remaining teeth | | **0.93 (0.88-0.98)** | 1.02 (0.98-1.07) | | 0.98 (0.94-1.03) |
|  |  |  |  | |  |
| AP = apical periodontitis; RF = root-filled; CI = confidence interval | | | |  |  |
| Values shown in bold text are statistically significant (p < 0.05: Logistic regression) | | | | | |
|  | |  |  | |  |

| **Supplementary Table 3.** The association between apical periodontitis and quality of root filling | | |
| --- | --- | --- |
| N = 756 |  |  |
|  |  | Odds ratio (95% CI) |
|  |  |  |
| Length of root filling | |  |
|  | Satisfactory | 1 |
|  | Short | **2.0 (1.4-2.8)** |
|  | Long | 1.2 (0.6-2.5) |
| Homogeneity of root filling | |  |
|  | Satisfactory | 1 |
|  | Unsatisfactory | **2.1 (1.5-2.9)** |
|  |  |  |
| CI = confidence interval  Values shown in bold text differ significantly from the | | |
| reference category (p < 0.05: Logistic regression) | | |
| Only adjusted (not crude) odds ratios are shown. | | |
|  | |  |
